# A hierarchical approach for building distributed quantum systems

**DOI:** 10.1038/s41598-022-18989-w

**Published:** 2022-09-14

**Authors:** Zohreh Davarzani, Mariam Zomorodi, Mahboobeh Houshmand

**Affiliations:** 1 Department of Computer Engineering, Ferdowsi University of Mashhad, Mashhad, Iran; 2 Department of Computer Engineering, Payame Noor University, Tehran, Iran; 3 Department of Computer Science, Faculty of Computer Science and Telecommunications, Cracow University of Technology, Krakow, Poland; 4 Department of Computer Engineering, Mashhad Branch, Islamic Azad University, Mashhad, Iran

**Keywords:** Quantum physics, Quantum information, Engineering, Mathematics and computing, Nanoscience and technology

## Abstract

In this paper, a multi-layer hierarchical architecture is proposed for distributing quantum computation. In a distributed quantum computing (DQC), different units or subsystems communicate by teleportation in order to transfer quantum information. Quantum teleportation requires classical and quantum resources and hence, it is essential to minimize the number of communications among these subsystems. To this end, a two-level hierarchical optimization method is proposed to distribute the qubits among different parts. In Level *I*, an integer linear programming model is presented to distribute a monolithic quantum system into *K* balanced partitions which results in the minimum number of non-local gates. When a qubit is teleported to a destination part, it can be used optimally by other gates without being teleported back to the destination part. In Level *II*, a data structure is proposed for quantum circuit and a recursive function is applied to minimize the number of teleportations. Experimental results show that the proposed approach outperforms the previous ones.

## Introduction

In the recent decade, the rapid growth of science and the engineering of quantum devices have led to the advancement of quantum computation from single isolated quantum devices toward multi-qubit processors^1^. As such, quantum computation has witnessed rapid growth with high performance in many areas. The standard approach in quantum computing is to design them as monolithic circuits.

Nowadays, quantum computing has many advantages over classical ones. One of them is that quantum computers can exponentially act better than classical ones for many computational problems^2^. Yet, due to implementation complexity, there are many challenges to design a large-scale quantum computer. The computing power of a quantum system increases exponentially with the number of embedded qubits^3^. A problem with greater qubits is more challenging for a quantum computer to solve.

Though advantageous, quantum computers have many shortcomings. One of these shortcomings is that the information of qubits may encounter errors before applying fault-tolerant approaches. This is due to the qubits, interconnected by the outside world which may lead to decoherence^4,5^ and when the number of qubits increases, the quantum information becomes more fragile and more susceptible to errors^6^. The error could also be due to the application of an operation on a quantum state^7^ which can be solved by separating qubits from their surroundings. As qubits establish the communication and some reading or writing operation, this solution is not reasonable. There are many solutions for these challenges. Physical implementations such as systems of trapped atomic ions can be accurately controlled and manipulated. A large variety of interactions and measurements of relevant observables can be engineered with high precision^8,9^. Also, superconducting qubit modality has been used to demonstrate prototype algorithms in the noisy quantum channel to have non-error-corrected qubits in quantum algorithms. Currently, this is one of the approaches for implementing medium and large-scale quantum devices and quantum coherent interactions with low noise and high controllability^10,11^. Another technology used to design a large-scale quantum system is photonic quantum computing. Quantum entanglement, teleportation, and quantum key distribution are derived from this technology because photons present a quantum system with low noise and high performance^12^.

One way to resolve these challenges is to divide quantum systems into some limited-capacity quantum systems, with qubits distributed on them, which is referred to as a Distributed Quantum System^13–15^.

### Distributed quantum circuit

Distributed quantum system consists of several independent quantum units with limited-capacity that appear as a single quantum system to the users. The units might be different from each other, in terms of hardware and software. For hardware limitations of each quantum unit, there is a connected graph called coupling map in each unit. The purpose of these limitations is to preserve and control qubits from decoherence and noise^7^.

Minimizing communications among quantum units of a distributed quantum system is very essential in reducing the cost of the whole system. On the other hand distribution of qubits among different subsystems leads to some non-local gates and to execute these non-local gates, it is essential to bring all qubits into a single subsystem. According to the no-cloning theorem, independent copies of qubits is not allowed in a quantum system. To this end, we can use teleportation protocol in order to move qubits between subsystems^16^. This protocol requires an entangled pair of qubits between two nodes in order to teleport the state of a qubit from one node to the other. This operation is expensive, and can lead to substantial latency due to the stochastic nature of underlying processes^17–19^. Therefore, minimizing communications among quantum units of a distributed quantum system is very essential in reducing the cost of the whole system. A proper distribution algorithm could decrease the communications between quantum units dramatically. With this in mind, this paper proposes the optimized distribution of quantum systems.

**Figure 1:**
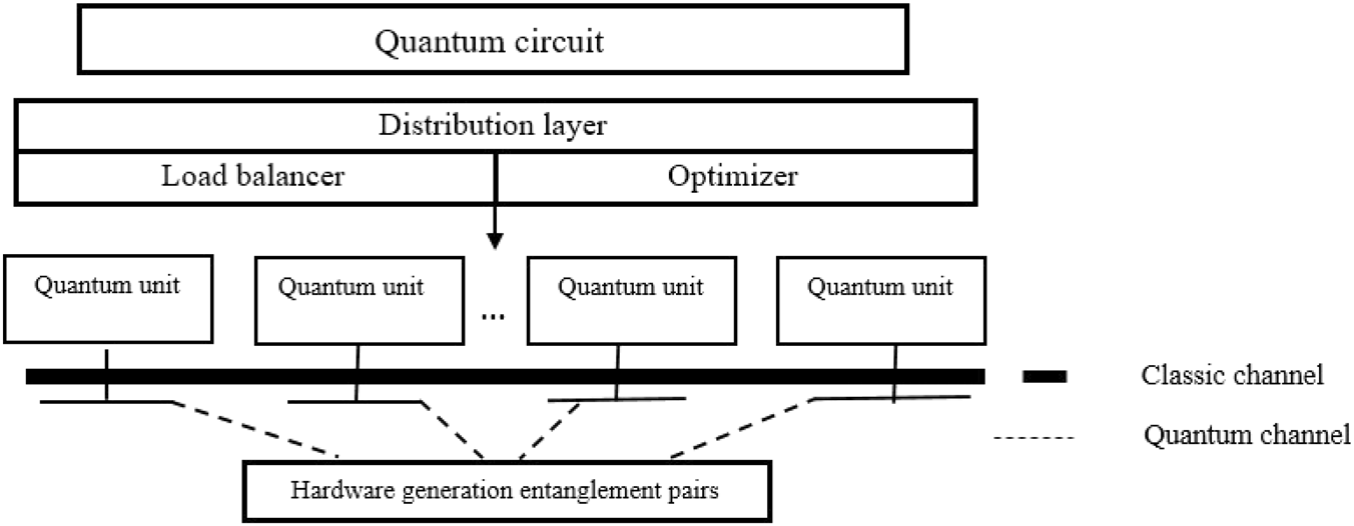
A multi-layer architecture of quantum circuit.

An abstraction of Distributed Quantum Computing is shown in Fig. 1 which is described through a set of (logical) layers, with the higher depending on the functionalities provided by the lower ones^7^. Starting from the top, there is the quantum algorithm in the form of quantum circuit. This algorithm is completely independent and unaware of logical and physical hardware constraints. In the second layer, there is a distribution algorithm. This algorithm implements the circuit of the previous layer in a distributed way. This layer consists of two parts called load balancer and optimizer, as follows:

- The qubits must be distributed well-balanced in some the limited-capacity quantum units. Therefore, a load balancing problem must be performed at this level.
- Non-local operations require qubits to communicate with qubits on other units. Hence, a teleportation protocol is needed for units to communicate. Minimizing the number of teleportations among these units is required at this level.

At the next level, quantum units communicate with each other via classical and quantum channels remotely. Both local and non-local operations can be executed at this level. The local operations execute on the qubits stored within the same quantum units and non-local operations execute on the qubits stored on different quantum units. As mention above, a quantum teleportation protocol is necessary for communication units with each other. This protocol consists of some phases such as, e.g. EPR pair generation, local operations, measurement and classical communications^15^. Each teleportation comprises two qubits stored on different units. These two qubits that are entangled together are called an entanglement pair. Each qubit of entanglement pairs is used to communicate a single qubit to another quantum device. Therefore, at the very bottom level, a hardware for generating entanglement pairs is required to communicate units with each other^20^. Each quantum device may have its own hardware to create an entanglement pair, or a separate device may generate this pair centrally^20^.

In this work, a two-level hierarchical optimization model is proposed to design a large-scale distributed quantum circuit. Hence, a monolithic quantum circuit is distributed to *K* quantum units. As such, minimizing communication between the *K* partitions is the objective. In Level I, an integer linear programming approach is proposed to distribute the qubits to *K* parts in a well-balanced manner. This minimizes the number of communications among these units. In this level, each non-local gate requires two teleportations because after a qubit is teleported to the destination, it is teleported back to its source. However, by teleporting one qubit of a non-local gate from the source to the destination, it may be used optimally in the destination by other non-local gates before being teleported back to its source. After the optimal utilization of the teleported-qubit, it can be returned to its source. Applying this concept can improve and minimize the number of teleportations. To this end, a recursive approach is proposed to consider for that in Level *II*. Therefore, through this hierarchical model, the required number of teleportations becomes fewer than the number of non-local gates.

The reminder of this paper is organized, as follows. “Related work” presents an overview of prior work. “The proposed algorithm” provides the proposed method in detail. Finally, “Experimental results” presents and discusses the experimental results.

## Related work

Distributed Quantum Computing (DQC) has been studied for many years. Scaling small-sized quantum systems to large-scale ones has been the main goal of these studies. The first study on DQC was reported in^21–23^. In that study, the author proposed the some quantum systems have physically located far from each other and sent the required information to a base station. He showed that the overall computation time is faster, in proportion to the number of such distributed quantum systems.

Moreover, DQC has been used in many applications. In^24^, the authors considered two black boxes as two quantum devices and they were prevented from communicating with others and designed trusted quantum cryptography to share a random key with security, based on quantum physics. A practical application for quantum machine learning (QML) was presented in^25^. In this application, a distributed secure quantum machine learning was considered for the classical client to delegate a remote quantum machine learning to the quantum server with data privacy. In^13^, two main approaches, i.e. teledata and telegate were discussed. In the telegate approach, teleporting gates enables them to be executed remotely without requiring qubits to be nearby. In teledat, qubits transfer their states to other systems without moving them physically.

Squash^26^ proposed a gate partition method by using METIS^27^ as the partitioning tool. Moghadam et al.^28^ used the min-cut approach presented in^29^ to divide the graph of the quantum circuit into smaller units. In^30^, the authors used the modified version of the graph partitioning algorithm of^31^ to minimize interaction between qubits. The authors of^32^ presented an architecture for DQC. They partitioned the quantum circuit by the multilevel k-way hypergraph algorithm presented in^33^.

Most recently, one strategy to scale up the number of qubits has been the quantum internet^34–36^. Quantum internet is a network of quantum systems which are able to interconnect with each other remotely via quantum and classical links. Distributed quantum computing is used in this network. In fact, the quantum internet is considered as a virtual machine consisting of several qubits and is scaled with the number of quantum devices in the network. This concept may indicate the possibility of an exponential speed-up quantum computing power^3,35^. In^3^, authors considered the challenges and open problems of Quantum internet design. They highlighted the differences between quantum and classical networks and discussed the critical research and challenges in designing quantum communication networks.

At first Yimsiriwattana et.al., in^37^ showed, for any contiguous non-local CNOT gates in which have common control qubit, the control line needs to be distributed only once, because it can be reused. This idea allows the number of communications reduce.

An automated method for distributing quantum circuits to *K* balanced partitions was investigated in^20^. They reduced the problem to hypergraph partitioning. Their algorithm consisted of two steps: pre and post processing for improving circuit distribution. It implements any number of contiguous non-local CNOT gates that execute on the same control qubit with target qubits in the same partition. They noted the consecutive non-local CNOT with the mentioned character can be executed with one teleportation.

Zomorodi et.al., presented several works^38–45^ for optimizing and partitioning of quantum circuits. Davarzani et.al.^38^ presented a dynamic programming approach to distribute a quantum circuit to *K* parts to minimize the number of communications. Their approach consisted of two steps. In the first step, the quantum circuit was converted into a bipartite graph. And in the next step, the bipartite graph was distributed to *K* parts by a dynamic programming approach. In that study, they tried to minimize the number of non-local CNOTs by converting the problem into minimum K-cut problems.

In another study^39^, an algorithm was proposed for DQC, consisting of two separated and long-distance quantum systems. They examined different configurations for the execution of non-local gates. Also, they ran their proposed algorithm for each configuration to reach the number of required teleleportations. The minimum number of communications was found among all the configurations. But, their proposed method had an exponential complexity.

An approach based on genetic algorithm has been used in^40^ to distribute a quantum circuit into two partitions. The main purpose of the algorithm was to determine which qubit of a non-local gate should be teleported to the other system and when the teleported qubit should be returned back to its home partition. Also, in our another work^41^, we presented a two-phase algorithm based on NSGA-II to bi-partition the qubits in the first phase and suggested two heuristics to optimize the number of non-local gates in the second phase. The authors in^42,44^ also discussed the issue of reducing communication cost in a distributed quantum circuit composing of up to three-qubit gates and presented a new heuristic method to solve it.

An automated Windows-Based method was proposed in^46^. In that study, the gate and qubit teleportation concept were combined with each other to minimize communication cost efficiently.

## The proposed algorithm

In this paper, we consider the problem of optimally distributing a given quantum circuit for evaluation over a set of subsystems and propose a two-level optimizer to reach a large-scale monolithic quantum circuit with the minimum number of required communications. The proposed method consists of two levels:

- Level I: In this step, the number of subsystems and the quantum circuit are given as inputs and the labelling $P:\{q_{i} |i\in \{1,...,N_{q}\}\}\rightarrow \{1,2,...,K\}$ of qubits to subsystems as output. Here, we partition the given circuit across distributed quantum circuits to reach near-balanced partitions of qubits. For this reason, an integer linear programming model is proposed to partition the quantum circuit into *K* parts. After distribution of qubits, some gates be non-local and each non-local gate requires two teleportation to the forward and backward qubits from source to destination units. Therefore, the number of communications is double equal to the number of non-local two-qubit gates obtained by this partitioning model.
- Level II: At this level, the obtained partitioning of Level *I* is considered as input and the minimum number of required teleportations is reached as output. As mentioned above, in the previous level, for each non-local gate, two teleportations are needed for forwarding and backwarding communications. When one of the qubits of non-local gates is teleported to the destination partition, more gates are executed by teleporting this qubit without the need of teleporting it back immediately. In this turn, the number of teleportations reduces. In this level, this idea is considered to optimize the number of teleportations. The details of these levels are as follows. Also, we use the notation of Table 1 in this paper.

**Table 1:** The notation of the proposed algorithm.

Notation	Description
$N_{g}$	Number of gates
$N_{q}$	Number of qubits
$run_{i}$	The status of *i*th gate which is executed (1) or not (0)
$P_{i}$	Partition number which $q_{i}$ is located on it
$N_{t}$	Number of teleportations
*K*	Number of partitions
$\omega$	Load-imbalance tolerance
$N_{non}$	Number of non-local gates
$N_{2qubit}$	Number of two-qubit gates

### Level I: the partitioning of quantum circuit

In this section, a *K*-way partitioning method is proposed to distribute a quantum circuit to *K* balanced partitions. This problem is a NP-hard problem and defined as follows:

#### Definition 1

Consider the undirected and weighted Graph $G = (V, E)$, where *V* denotes the set of *n* vertices and *E* the set of edges. The balanced graph partitioning problem takes the Graph *G*(*V*, *E*), Parameter *K* as the number of partitions and Parameter $\omega$ known as the load balance tolerance as inputs. We wish to partition the graph into *K* balanced disjoint parts or sub-graphs $(V_{1}, V_{2},...,V_{K})$ so that $V={V_{1} \cup V_{2}\cup ... \cup V_{K }}$. Two criteria must be satisfied as follows:

- Minimum number of cuts: the number of cuts among all the different sub-graphs is minimized as Eq. (1):
  1 $$\begin{aligned} min \sum \limits _{k=1}^{K} \sum \limits _{l=k+1}^K \sum \limits _{v_{1}\in V_{k},v_{2}\in V_{l}} C({v_{1},v_{2}}) \end{aligned}$$
  where $C({v_{1},v_{2}})$ is the weight of edge $(v_{1},v_{2})$.
- Load-balance: for all $k=1,2,...,K$:
  2 $$\begin{aligned} |V_k|\le \frac{(1+\omega )|V|}{K} \end{aligned}$$

As a combinational problem, many heuristic approaches are mostly used to the graph partitioning to the need acceptable computation time. We reduced the problem of balanced distribution of quantum circuit to the problem of balanced graph partitioning so that qubits and gates are the nodes and edges in graph respectively. We proposed an integer linear programming model for $K-way$ partitioning of quantum circuits. Let the quantum circuit consist of two sets, i.e. $Q=\{q_{i} |i \in {1,...,N_{q}}\}$ and ${\mathcal {G}} =\{g_{j} |j\in {1,...,N_{2qubit}}\}$ where set ${\mathcal {G}}$ is the set of two-qubit gates. Each $g_j$ operates on two Qubits $q_{i_{1}}$ and $q_{i_{2}}$ and has been shown as $g_j(q_{i_{1}},q_{i_{2}})$. The binary variables of the proposed mathematical model are as Eqs. (3) and (4):

3 $$\begin{aligned} f_{j}= & {} {\left\{ \begin{array}{ll} 1&{} \text {if }g_j\hbox { is\, a\, non-local\, gate}\\ 0&{} \text {otherwise} \end{array}\right. } \end{aligned}$$

4 $$\begin{aligned} p_{i,k}= & {} {\left\{ \begin{array}{ll} 1&{} \text {if }q_{i}\hbox { has\, been\, located \,on \,Part\, }k\\ 0&{} \text {otherwise} \end{array}\right. } \end{aligned}$$

The binary variable $f_{j}$ is set to one when a two-qubit Gate $g_{j}(i_1,i_2)$ is a non-local gate and Qubits $i_1$ and $i_2$ have been located on the different parts and zero otherwise (local gate). Also the binary Variable $p_{i,k}$ is determined whether $q_{i}$ be located to the Part *k* or not. The proposed model is given in Eqs. (5) to (9):

5 $$\begin{aligned}&\min \sum \limits _{j=1}^{N_{2qubit}} f_{j} \end{aligned}$$

6 $$\begin{aligned}&\sum \limits _{i=1}^{N_{q}} p_{i,k} \le (1+\omega )|Q| /K \quad \forall k=1,...,K \end{aligned}$$

7 $$\begin{aligned}&\sum \limits _{k=1}^{K} p_{i,k}=1 \quad \forall i=1,...,N_q \end{aligned}$$

8 $$\begin{aligned}&f_{j}\ge p_{i_2,k}-p_{i_1,k} \quad \forall k=1,...,K, \quad g_j(i_{1},i_{2}) \in \mathcal {G} \end{aligned}$$

9 $$\begin{aligned}&f_{j}\ge p_{i_1,k}-p_{i_2,k} \quad \forall k=1,...,K, \quad g_j(i_{1},i_{2}) \in \mathcal {G} \end{aligned}$$

S.t $$f_{j} \in \{0,1\}, p_{i_1,k}\in \{0,1\} \quad \forall i=1,...N_{q}, j=1,...,N_{2qubit}, k=1,...,K$$

Equation (5) determines the objective function. In this problem, the number of non-local gates is considered as the objective function. Load balancing criteria is considered in Eq. (6). Equation (7) ensures that a qubit is assigned to exactly one unit. Equations (8) and (9) guarantee that non-local gates are correctly accounted.

The proposed model distributes the quantum circuit into *K* balanced units. This distribution involves mapping the qubits of circuit into *K* subsystems. The output should be a labelling $f: Q \rightarrow \{1,...,K\}$ of qubits to satisfying two criteria given in Eqs. (1) and (2). This function maps the qubits to a set of labels $P=\{p_1,p_2,...,p_K\}$. These labels are as input of Level *II*.

### Level II: the optimization level

After partitioning of Level I, qubits are distributed into *K* units according to the obtained labeling of the previous level. As stated earlier, each non-local gate needs two teleportations for executing. It is clear that in many cases, teleporting a qubit from its source partition to the destination partition, known as the migrated qubit, makes it optimally available to use by other gates without the need to teleport it back to its own partition. After that, the migrated qubit is teleported back to its home partition. At this level, we propose a recursive approach to implement this issue and minimize the total number of teleportations.

In this level, we present a data structure for representing quantum circuits. This structure is a two-dimensional matrix called $C_{N_{q}\times N_{g}}$ with $N_q$ rows and $N_g$ columns and defined as follows:

- Qubits are located on the rows and numbered from one to $N_{q}$, where the *i*th row indicates Qubit $q_{i}$.
- Gates are located on the columns and are numbered in the order of their executions in the quantum circuit.

Element $C_{i,j} \quad (1\le i\le N_{q}, 1\le j\le N_{g})$ consists of two components: (*index*, *label*). *index* is the qubit that communicates with *i*th qubit in *j*th gate and *label* is the type of this qubit in which is ‘control’ or ‘target’ in two-qubit gates or ‘non’ in one-qubit gates. These elements are constructed as follows:

- For each two-qubit gate $g_{i} (q_{t},q_{c})$, $C_{t,i}=(q_c,$‘*c*’) and $C_{c,i}=(q_t,$‘*t*’).
- For each one-qubit gate $g_{i} (q_{j})$, $C_{j,i}=(q_j,non)$.
- Other elements are quantified by zero.

**Figure 2:**
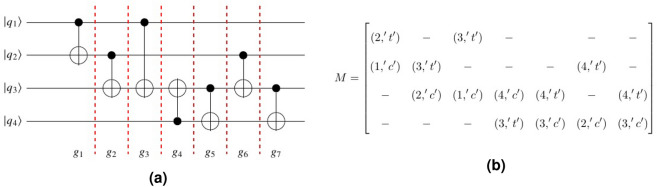
(**a**) A sample of quantum circuit, (**b**) and its corresponding matrix.

For example, we consider a quantum circuit with 4 qubits and 7 gates in Fig. 2a so that its corresponding matrix is given in Fig. 2b.
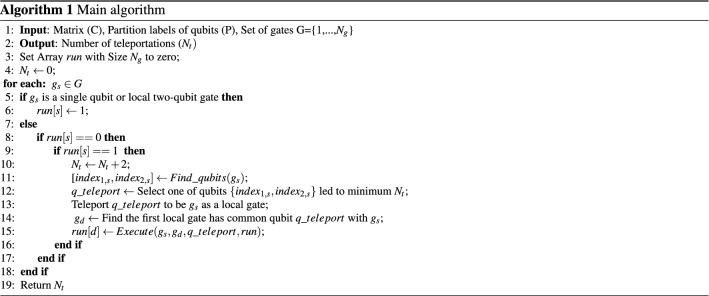

Algorithm I presents the main algorithm. In this algorithm, we used an array called *run* with Size $N_{g}$ in which *run*[*i*] indicates the status of the *i*th gate in which it has/has not been executed. The algorithm starts from the first gate or column of *C* (Index *s*). It may indicate one of the following three conditions:

- Column *s* indicates a local two-qubit gate.
- Column *s* is a one-qubit gate.
- Column *s* indicates a non-local gate.

In the first two cases, no teleportation is required and these gates are executed and *run*[*s*] is set to one (Lines 5–6 of the main algorithm). Otherwise, Gate $g_s$ is a non-local gate and a teleportation is required for the executing of $g_s$ . Then the teleportation cost is increased by two (Line 10) in which one additional teleportation must be accounted for transferring the qubit back to its source part. Then Function $Find\_qubits(g_s)$ finds two qubits of Gate $g_{s}$ called Qubits $index_{1,s}$ and $index_{2,s}$. One of these qubits called $q\_teleport$ which led to the minimum number of teleportations, is selected (Line 12). This qubit is teleported from its own part to the destination to execute gate $g_s$. The algorithm tracks the whole circuit to find the gate that can be executed without returning $q\_teleport$ to its source. This means the teleported qubit is optimally used by the other gates which require $q\_teleport$ and can be executed.

**Figure 3:**
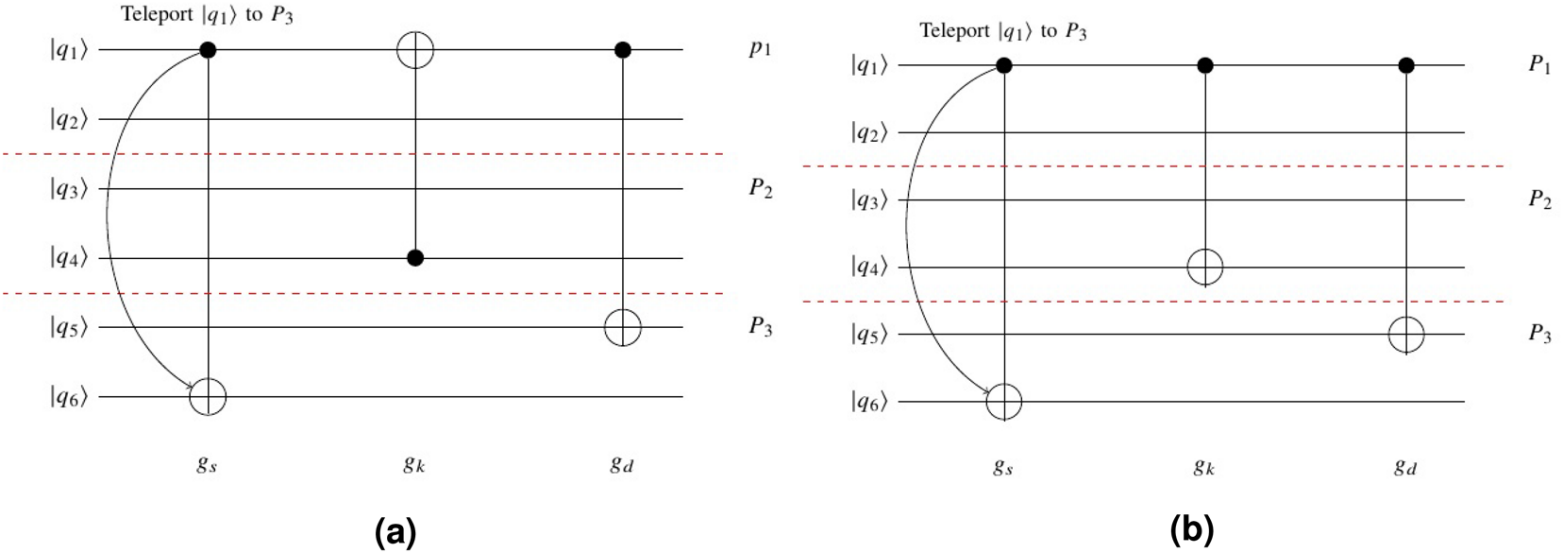
(**a**) By teleporting $q_{1}$ to $P_{3}$, Gate $g_{d}$ cannot be executed. (**b**) By teleporting Qubit $q_{1}$ to $P_{3}$, Gate $g_{d}$ can be executed.

Let Gate $g_{d}$ in Column *d* be the first local two-qubit gate called $g_{d} (index_{1,d},index_{2,d})$ in whis has common Qubit $q\_teleport$ with Gate $g_s$. This gate must be considered whether it can be executed or not. Function $Execute(g_{s},g_{d},q\_teleport, run)$ is a recursive function and considers by teleporting $q\_teleport$, Gate $g_{d}$ can be executed or not. This function is shown in Algorithm *II*. Three states may occur in this function as follows:

- The function returns *False* when there is at least a non-executed and non-local gate between $g_{s}$ and $g_{d}$ which has not been executed before $g_{d}$ and the execution of $g_d$ depends on it. Let Column $k (s< k <d)$ as $g_{k} (index_{1,k},index_{2,k})$ be the first non-executed and non-local gate before Column *d* in which has a common qubit with Gate $g_d$. This column has two non-zero rows $index_{1,d}$ and $index_{2,d}$. This function returns *False* ( Line 11 of Algorithm II) and stops due to the following condition:
  10 $$\begin{aligned}& index_{i,k} = index_{j,d} \quad \& \& \\& P_{index_{\{1,2\} - i,k}} \ne P_{index_{\{1,2\}-j,d}} \quad \& \& \\& \biggl (C[k,index_{i,k}].label \ne C[d,index_{j,d}].label\quad \Vert \\& C[k,index_{i,k}].label = C[d,index_{j,d}].label==`t`\biggr ) \exists i,j \in \{1,2\} \end{aligned}$$
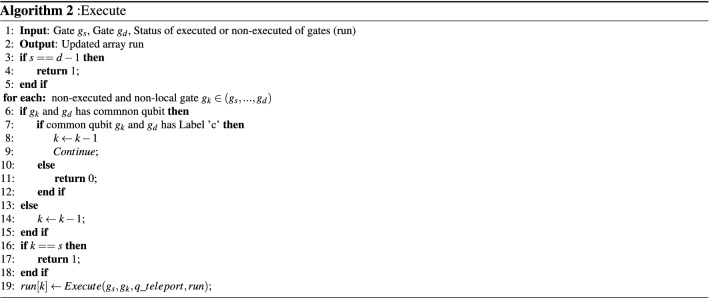
  Equation (10) indicates one of the qubits of $g_{k}$ is the same as the qubits of $g_{d}$ with a different label or the same Label ‘*t*’ and another qubit of $g_{k}$ and $g_d$ has been located on the different partitions. In this case, another teleportation is required to execute $g_{k}$ and the function returns *False*, as a result. Figure 3a shows this concept. In this example, $q_{1}$ is teleported from $P_{1}$ to $P_{3}$ to execute $g_s$. By this teleporting, executing of Gate $g_{d}$ should be considered by Function *Execute*. This function finds non-executed and non-local Gate $g_{k}$ before Gate $g_{d}$ in which have common qubit $q_{1}$ with a different label. Since execution of Gate $g_d$ depends on execution of Gate $g_{k}$ and Gate $g_k$ is a non-local gate, Then Gate $g_d$ cannot execute and Function *Execute* returns *False*.
  Sometimes Gate $g_{k}$ may be a non-local gate in which has a common qubit with Gate $g_{d}$ with Label ‘*c*’. In this case, the execution of Gate $g_{d}$ is independent the execution of Gate $g_{k}$. This in turn, non-execution Gate $g_{k}$ prevented to execution of Gate $g_d$ and the execution of other previous gates of $g_{d}$ are considered (Lines 7–9) . Equation (11) indicates this state.
  11 $$\begin{aligned} index_{i,k}= index_{j,d}\quad \& \& ({C[ {k,inpu{t_{i,k}}} ].label =C[{d,inpu{t_{j,d}}} ].label = `c`} ) \quad \exists i,j \in \{1,2\} \end{aligned}$$
  This state is shown in Fig. 3b.
- There are no gates between Gate $g_{s}$ and Gate $g_{d}$ to prevent the execution of Gate $g_d$. In this case, this function returns *True* (Lines 13–14).
- If Gate $g_{k}$ does not meet any of the conditions of Eqs. (10) and (11), Function $Execute(g_s,g_k,q\_teleport,run)$ is called recursively to consider if $g_{k}$ is executed or not (Line 19).

**Figure 4:**
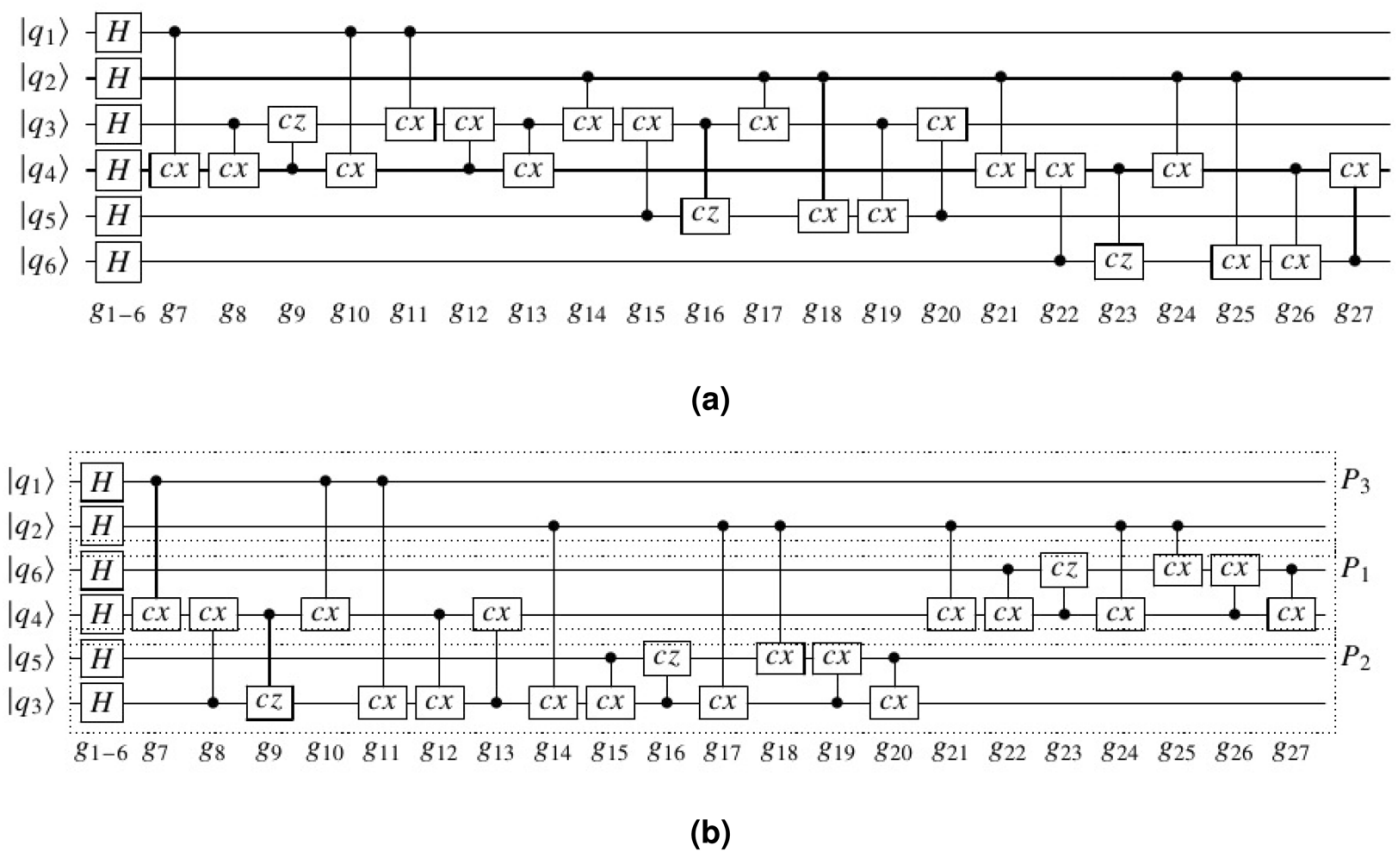
(**a**) Circuit 2–4 dec. (**b**) The obtained circuit from applying Level I. The gate $g_{i},i=\{7,8,9,10,11,12,13,14,17,18,21,24,25\}$ are non-local gates.

The proposed method is explained by an example. Figure 4a shows quantum circuit 2–4 dec given from Revlib^47^. This circuit consists of six qubits and 27 gates. Our algorithm distributes this circuit into three partitions each containing two qubits. At first, Level I of proposed method distributes this circuit as shown in Fig. 4b and Array *P* is quantified as [3,3,2,1,2,1]. In this level, the number of non-local gates is obtained 13 and then total number of communications is set to 26. Table 2 demonstrates the steps of Level *II* of our method on this circuit. In this table, $g_s$, status of $g_s$ (one-qubit/ local/ non-local gate) and qubit which is teleported ($q\_teleport$) are given in Column 2. In Column 3, the partition that $q\_{teleport}$ is teleported to it (destination partition), $g_d$ and the partition that $q\_teleport$ is teleported back to it (source partition) are depicted respectively. Also the Array *run* that indicates *i*-th gate is executed or not is shown in Column 4 and array *P* is given in the last column. The steps of Level *II* is as following:

- Step 1: $g_1$ to $g_6$ are one-qubit gates and no teleportation is required. Then $run[i]=1,\{i=1,...,6\}$.
- Step 2: $g_7(q_1,q_4)$ is a non-local gate and $q_1$ is teleported to $P_1$. $g_{10}$ is the first gate which has common qubit $q_1$ with $g_7$. Since $g_{10}$ is dependent to $g_9$ and $run[9]=0$, $g_{10}$ could not be executed. Therefor $g_7$ is only executed and $run[7]=1$. Then $q_1$ is teleported back to $P_3$;
- Step 3: $g_8(q_3,q_4)$ is a non-local gate and $q_3$ is teleported to $P_1$. $g_9$ is the first gate which has common qubit $q_3$ with $g_8$. Then $run[i]=1,i=\{8,9\}$. Other gates could not be executed and $q_3$ is teleported back to its source partition ($P_2$).
- Step 4: $g_{10}(q_1,q_4)$ is a non-local gate and $q_1$ is teleported to $P_1$. Any gate has common qubit with $g_{10}$. Then $run[10]=1$ and $q_1$ is teleported back to $P_3$.
- Step 5: $g_{11}(q_1,q_3)$ is a non-local gate and $q_1$ is teleported to $P_2$. Any gate has common qubit with $g_{11}$. Then $run[11]=1$ and $q_1$ is teleported back to $P_3$.
- Step 6: $g_{12}(q_3,q_4)$ is a non-local gate and $q_4$ is teleported to $P_2$. $g_{13}$ is the first gate which has common qubit $q_4$ with $g_{12}$. Therefor $g_{13}$ is only executed and $run[i]=1,i=\{12,13\}$. Then $q_4$ is teleported back to $P_1$.
- Step 7: $g_{14}(q_2,q_3)$ is a non-local gate and $q_2$ is teleported to $P_2$. $g_{18}$ and $g_{17}$ have common qubit $q_2$ with $g_{14}$. These gates are dependent to $g_{15}$ and $g_{16}$ which are local gates and could be executed. Then $run[i]=1, i=\{14,...,18\}$ and $q_2$ is teleported back to $P_3$.
- Steps 8 and 9: $g_{19}$ and $g_{20}$ are local gates and executed. Then $run[i]=1,i=\{19,20\}$.
- Step 10: $g_{21}(q_2,q_4)$ is a non-local gate and $q_2$ is teleported to $P_1$. Then $run[i]=1,i=\{21,...25\}$. Then $q_2$ is teleported back to $P_3$.
- Steps 11 and 12: $g_{26}$ and $g_{27}$ are local gates and are executed. Then $run[i]=1,i=\{26,27\}$.

As shown above, each of Steps 1, 2, 3, 4, 5, 6 and 9 require to two teleportations. Then the total number of teleportations is 14 for this circuit.

**Table 2:**
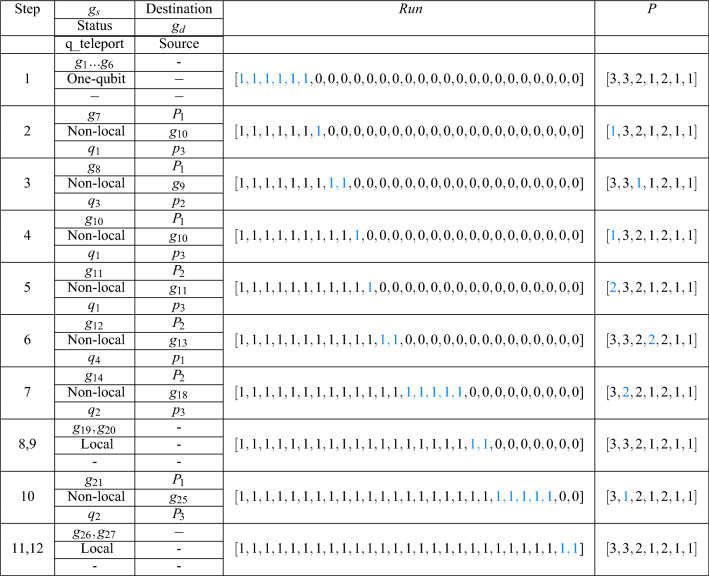
The steps of distribution Circuit 2-4dec into 3 partitions with 7 qubits and 27 gates.

## Experimental results

We implemented our method in MATLAB on a Core i7 CPU operating at 1.8 GHz with 8 GB of memory. We used many circuits to compare the performance of the proposed method with previous approaches: that of^39^, the dynamic programming approach of^38^, the evolutionary algorithm of^40^, the automated approach of^20^ and the windows-based method of^46^. The benchmark circuits are given from^48^ (the circuits from 1 to10), Revlib^47^ (the circuits from 11 to 15 and 26 to 31), some quantum error-correction encoding circuits^49^(the circuits from 16 to 25) and n-qubit Quantum Fourier Transform circuits (QFT)^50^ where $n \in \{16, 32, 64, 128, 256\}$. The benchmark circuits include some of the gates of the gate library synthesized following the method in^51^. In this paper CNOT, CZ and one qubit gates are considered as the gate library.

To put the quality of results into perspective, the standard deviation criterion is employed as Eq. (12):

12 $$\begin{aligned} Dev=\frac{T_{ap}-T_{best}}{T_{best}}*100 \end{aligned}$$

Where $T_{best}$ is the best number of teleportations obtained among all of approaches and $T_{ap}$ is the obtained number of teleportations of approach that we compare ours to.

First, Table 3 shows the number of teleportations in comparison with the windows-based approach of^46^. In this table, the number of qubits, gates and partitions are given in Columns 3, 4 and 5. Also Columns 6, 7, 8 and 9 report the number of teleportations and *Dev* of the proposed method and method^46^, respectively. As shown in this table, except Circuits 2-4dec, Cycle17_3, Ham15-D3, Ham7_106 and Parity247, *Dev* for our approach have zero value and demonstrate the proposed method outperformed that of^46^ to reach minimum number of teleportations in these circuits.

**Table 3:** The number of teleportations $(N_t)$ and *Dev* of the proposed algorithm in comparison with the method of^46^.

#	Benchmark	$N_{q}$	$N_g$	K	Proposed method		Method of^46^	
					$N_t$	Dev	$N_t$	Dev
1	2of5-D1	6	104	2	40	0	40	0
2	2-4dec	6	21	3	14	27	11	0
3	6sym	10	72	2	14	0	16	14
4	9sym	12	108	3	22	0	36	63
5	8bitadder	24	338	6	46	0	84	82
6	Cycle17_3	20	5861	3	2262	11	2028	0
7	Ham15-D3	15	220	4	144	29	101	0
8	Hwb50	56	6430	4	900	0	1301	44
9	Hwb100	107	6430	4	2014	0	2513	24
10	Rd32 272	5	5	2	4	0	6	50
11	Ham7_106	7	49	4	34	13	30	0
12	Rd53_139	8	49	2	8	0	12	50
13	Rd53_311	13	104	3	16	0	22	37
14	Parity 247	17	16	3	6	50	4	0
15	Adder16_174	49	200	3	6	0	9	50

**Figure 5:**
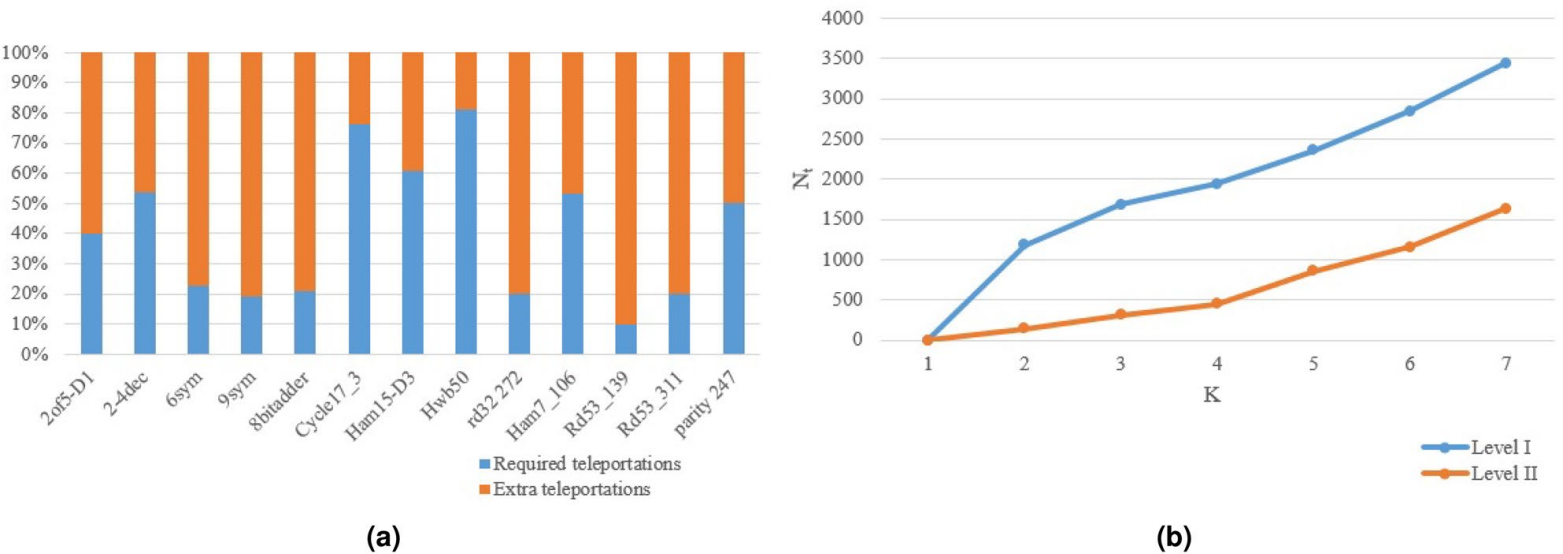
(**a**) Percentage of required number of teleportations to extra teleportations on Circuits 1 to 15. (**b**) The effect of the number of units on number of communications of Level I and II on Circuit Hwb50.

First, it is important to demonstrate how applying Level *II* to the partitioning of Level I improves the number of communication. As mentioned before in that section, in Level *I* of the proposed algorithm, two teleportations are needed to execute each non-local gate because after a qubit is teleported to the destination home, it is teleported back to its source. Also, the proposed algorithm on level II allowed to teleported qubit to used optimally in the destination home. Therefore, it can save many number of quantum teleportations. Figure 5a shows the effectiveness of applying Level II to Level I to decrease the number of teleportations for Circuits 1 to 15. As can be seen, the bottom bar (the blue bar) indicates the required number of communications after applying Level II to these benchmarks and the top bar (the orange bar) indicates extra teleportations without applying Level II. As shown in this figure, in all of cases, over 70% of non-local gates could be implemented locally and the Level *II* reduces the number of teleportations to less than half in all of the samples.

In another test, we considered the impact of the number of subsystems on the number of teleportations. A near-balanced distribution of qubits over more quantum circuits requires more communications. Figure 5b demonstrates the effect of the number of units (*K*) on the required number of teleportations on Circuit Hwb50 with 56 qubits and 6430 gates. In this figure, qubits are distributed across {2, 3,..., 7} units. As shown in this figure, an increase in the number of partitions used to distribute qubits requires more communications among them and a large number of teleportations is used. Lines Blue and Orange show the obtained number of teleportations before and after applying Level *II* to Circuit Hwb50, respectively.

Second, we tested our method on another benchmark (numbered 16–25) and compared with method of^46^. These results are demonstrated in Table 4. The best obtained results are marked in bold. Except of three circuits, our approach has outperformed in comparison to^46^.

**Table 4:** Comparison the number of teleportations of proposed method ($N_t$) with proposed approach of^46^ on Circuits 16 to 25.

#	Benchmark	$N_{q}$	Depth	*K*	$N_t$	$N_t$^46^
16	[[10-3-3]]	10	25	2	**10**	13
17	[[16-3-5]]	16	43	4	62	**48**
18	[[21-1-7]]	21	58	3	60	**56**
19	[[24-3-7]]	24	84	4	**86**	101
20	[[25-1-9]]	25	83	5	**72**	88
21	[[27-1-9]]	27	110	4	**96**	98
22	[[31-1-16]]	31	149	4	**126**	138
23	[[33-1-9]]	33	153	4	184	**138**
24	[[35-1-10]]	35	126	4	**116**	120
25	[[40-3-10]]	40	172	4	**162**	184

**Figure 6:**
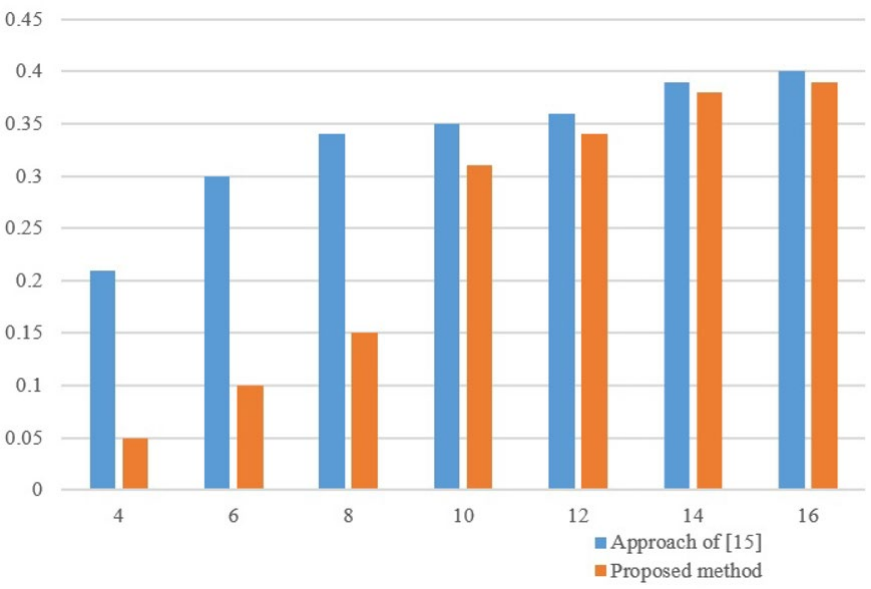
The proportion of the required teleportations over the total two-qubit gates when QFT circuit is distributed across 4,6,8,...16 quantum devices in comparison with^20^.

Third, we ran our method on QFT circuit in comparison with the method of^20^. We distributed the quantum circuit across {4,6,8,...16} quantum devices. Also, the $N_q$ and $N_g$ are 201 and 19900, respectively. Figure 6 shows the proportion of the number of teleportations over the total number of two-qubit gates for our approach and approach of^20^. As shown in this figure, this ratio grows by increasing the number of partitions. Also our approach has acted better than method^20^ in all cases in terms of the ratio between the number of teleportations and the number of two qubit gates. Since, the proposed approach considers all of the configurations to execute more non-local gates, it found the minimum number of communications in comparison with the approach of^20^ in which they implemented a group of non-local gates with a common control qubit only. As shown in this figure, when QFT is distributed in $K=\{4,6,8\}$, the number of teleportations obtained by the proposed approach have many differences from the approach of^20^, but two methods acted almost identically for $K=\{10,12,14,16\}$.

**Figure 7:**
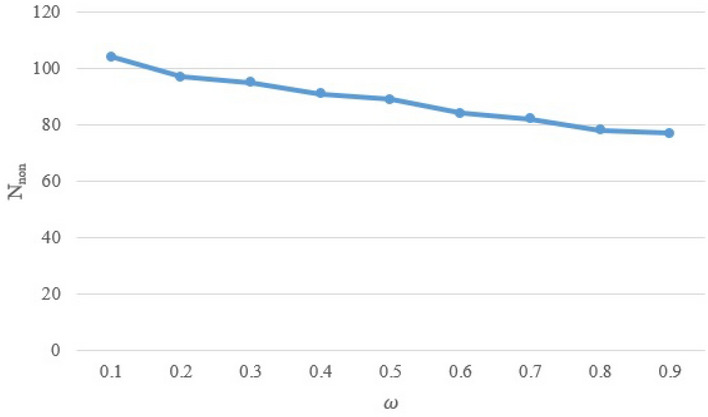
The effective of load-balance tolerance ($\omega$) on $N_{non}$.

In another test, we demonstrate the effectiveness of load-balance tolerance ($\omega$) on the number of non-local gates in Level I. Figure 7 shows the number of non-local gates for various $\omega =\{0.1,...,0.9\}$ on one sample circuit. As shown in this figure, $N_{non}$ is reduced by increasing the load-balance tolerance. According to Eq. (6), when Factor $\omega$ increases, the qubits that have many communications with each other, are located in the same partition. Therefore, the number of non-local gates is reduced.

**Table 5:** The teleportation cost of the proposed method ($N_t$) on Circuits 26 to 31 in comparison with of^38–40^.

#	Benchmark	$N_{q}$	# gates	*K*	$N_t$^38^	$N_t$^39^	$N_t$^40^	$N_t$(p)
26	Pariaty_247	17	16	2	2	2	2	2
27	Sym9_147	12	108	2	8	N.A.	48	6
28	Flip_flop	8	30	3	8	N.A.	N.A.	8
29	Alu_primitive	6	21	2	6	18	20	2
30	Alu_primitive_opt	6	21	2	6	10	10	4
31	Figure 4 of^39^	4	7	2	2	4	4	2

Another set of the test samples was taken from Revlib to compare the proposed method with the other approaches^38–40^ such as: Alu_primitive, Parity, Flip_flop, Sym9_147 (the circuits 26 to 31). The number of qubits, gates and partitions are given in Columns 2, 3 and 4 of Table 5 respectively. Also Columns 5, 6 and 7 report the number of teleportations of^38–40^ too. The last column shows the obtained number of the teleportations of the proposed approach. As can be seen, the proposed method outperformed the other approaches.

## Conclusion

In this paper, a two-level hierarchical architecture of distributed quantum computing was proposed to build large quantum systems in which the number of communications among quantum subsystems is minimized. In the first level, an integer linear programming model was proposed to distribute the qubits to *K* balanced subsystems. In the second level, we presented a new data structure for representing quantum circuits. Also, according to the partitioning of the first level, when one of the qubits of a non-local gate is teleported from its source subsystem to the destination, it is used optimally by other gates in the destination subsystem before being teleported back to its own subsystem. Moreover, we proposed a recursive method to optimize the number of teleportations. Finally, we ran the proposed method on the different benchmarks and showed that it produces better results in comparison with the previous ones.
